# Artificial intelligence-based classification of cardiac autonomic neuropathy from retinal fundus images in patients with diabetes: The Silesia Diabetes Heart Study

**DOI:** 10.1186/s12933-024-02367-z

**Published:** 2024-08-10

**Authors:** Katarzyna Nabrdalik, Krzysztof Irlik, Yanda Meng, Hanna Kwiendacz, Julia Piaśnik, Mirela Hendel, Paweł Ignacy, Justyna Kulpa, Kamil Kegler, Mikołaj Herba, Sylwia Boczek, Effendy Bin Hashim, Zhuangzhi Gao, Janusz Gumprecht, Yalin Zheng, Gregory Y. H. Lip, Uazman Alam

**Affiliations:** 1 Department of Internal Medicine, Diabetology and Nephrology, Faculty of Medical Sciences in Zabrze, Medical University of Silesia, Katowice, Poland; 2 Liverpool Centre for Cardiovascular Science at University of Liverpool, Liverpool John Moores University and Liverpool Heart and Chest Hospital, Liverpool, UK; 3 Student’s Scientific Association at the Department of Internal Medicine, Diabetology and Nephrology, Faculty of Medical Sciences in Zabrze, Medical University of Silesia, Katowice, Poland; 4 Doctoral School, Department of Internal Medicine, Diabetology and Nephrology, Faculty of Medical Sciences in Zabrze, Medical University of Silesia, Katowice, Poland; 5 Department of Eye and Vision Science, Institute of Life Course and Medical Sciences, University of Liverpool, Liverpool, UK; 6 Paul’s Eye Unit, Royal Liverpool University Hospital, Liverpool, UK; 7 Danish Center for Health Services Research, Department of Clinical Medicine, Aalborg University, Aalborg, Denmark; 8 Diabetes & Endocrinology Research and Pain Research Institute, Institute of Life Course and Medical Sciences, University of Liverpool and Liverpool University Hospital NHS Foundation Trust, Liverpool, UK

**Keywords:** Cardiac autonomic neuropathy, Artificial intelligence, Retinal imaging, Deep learning, Cardiovascular risk assessment

## Abstract

**Background:**

Cardiac autonomic neuropathy (CAN) in diabetes mellitus (DM) is independently associated with cardiovascular (CV) events and CV death. Diagnosis of this complication of DM is time-consuming and not routinely performed in the clinical practice, in contrast to fundus retinal imaging which is accessible and routinely performed. Whether artificial intelligence (AI) utilizing retinal images collected through diabetic eye screening can provide an efficient diagnostic method for CAN is unknown.

**Methods:**

This was a single center, observational study in a cohort of patients with DM as a part of the Cardiovascular Disease in Patients with Diabetes: The Silesia Diabetes-Heart Project (NCT05626413). To diagnose CAN, we used standard CV autonomic reflex tests. In this analysis we implemented AI-based deep learning techniques with non-mydriatic 5-field color fundus imaging to identify patients with CAN. Two experiments have been developed utilizing Multiple Instance Learning and primarily ResNet 18 as the backbone network. Models underwent training and validation prior to testing on an unseen image set.

**Results:**

In an analysis of 2275 retinal images from 229 patients, the ResNet 18 backbone model demonstrated robust diagnostic capabilities in the binary classification of CAN, correctly identifying 93% of CAN cases and 89% of non-CAN cases within the test set. The model achieved an area under the receiver operating characteristic curve (AUCROC) of 0.87 (95% CI 0.74–0.97). For distinguishing between definite or severe stages of CAN (dsCAN), the ResNet 18 model accurately classified 78% of dsCAN cases and 93% of cases without dsCAN, with an AUCROC of 0.94 (95% CI 0.86–1.00). An alternate backbone model, ResWide 50, showed enhanced sensitivity at 89% for dsCAN, but with a marginally lower AUCROC of 0.91 (95% CI 0.73–1.00).

**Conclusions:**

AI-based algorithms utilising retinal images can differentiate with high accuracy patients with CAN. AI analysis of fundus images to detect CAN may be implemented in routine clinical practice to identify patients at the highest CV risk.

**Trial registration:**

This is a part of the Silesia Diabetes-Heart Project (Clinical-Trials.gov Identifier: NCT05626413).

**Graphical Abstract:**

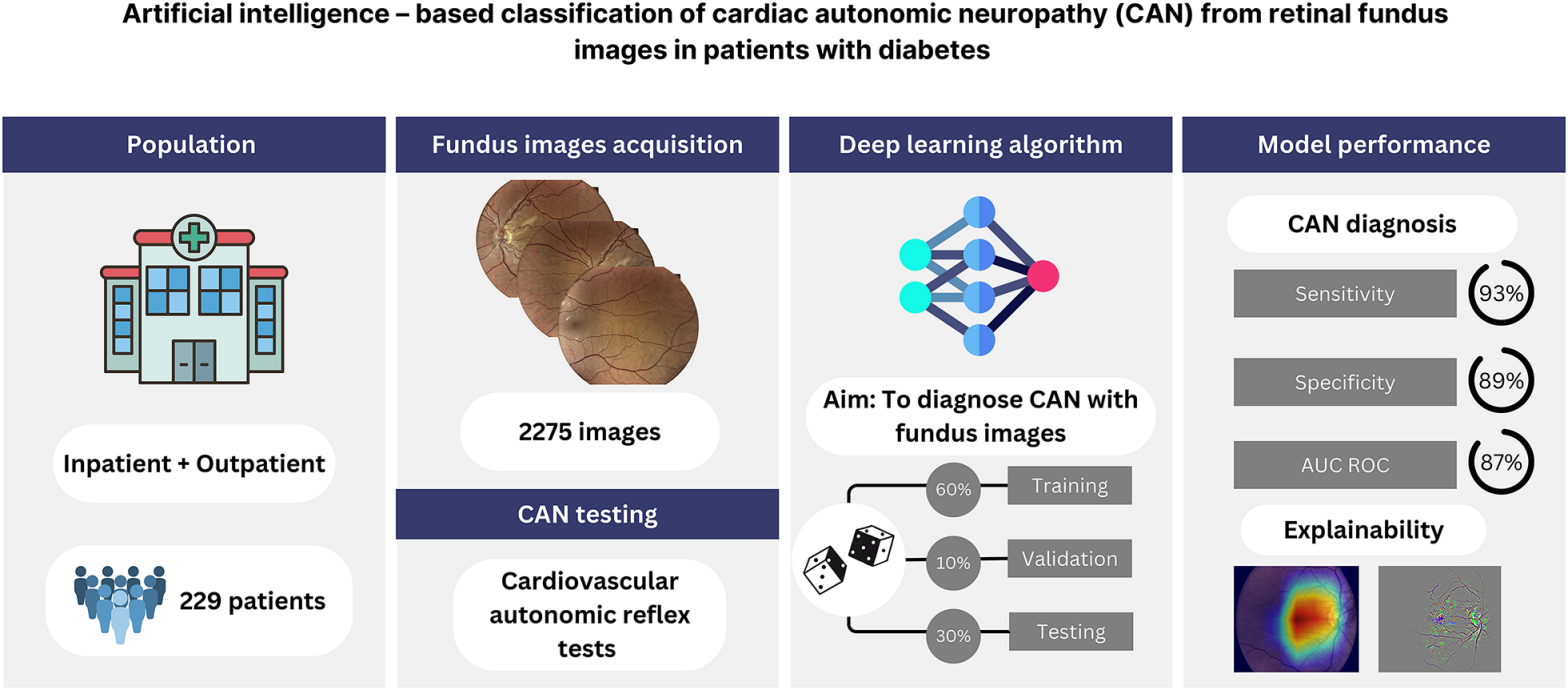

**Supplementary Information:**

The online version contains supplementary material available at 10.1186/s12933-024-02367-z.

## Introduction

The International Diabetes Federation estimates that globally ~ 10.5% of adults have diabetes mellitus (DM) which is predicted to more than double by 2050 [1]. Cardiac autonomic neuropathy (CAN) is a common but yet underdiagnosed microvascular complication of DM [2]. The prevalence of CAN increases with DM duration in both type 1 DM (T1DM) and type 2 DM (T2DM) with up to 30% [2, 3] and 60% after 20 years being diagnosed and after 15 years of DM duration [4] respectively.

Early detection of CAN is pivotal due to its role as an independent risk factor for CV (cardiovascular) mortality, arrhythmias, silent myocardial ischemia, with ~ 3 × relative risk for CV events and mortality [5, 6]. Current diagnostic modalities for CAN are limited, with the majority being labour-intensive cardiac autonomic reflex tests [7]. These tests, given their intricate nature, are not routinely employed in standard clinical practice. Hence, in clinical practice the majority of diagnoses occur with the development of symptoms which indicates advanced end-stage CAN with almost total autonomic denervation [7].

Artificial intelligence (AI) is becoming more widely used for diagnosis with recent advances demonstrating connections between different patients' phenotypes, features, parameters and body examination images [8]. Recently, we have developed AI-based deep learning algorithm utilizing corneal confocal microscopy images of the subbasal nerve plexus to detect the peripheral neuropathy with excellent accuracy [9]. AI-enabled retinal imaging can predict circulatory mortality, myocardial infarction and stroke [10] and more recently neurological disorders [11].

Colour fundus photography of the retina is easy to perform and used in routine annual diabetic retinopathy (DR) screening. However, retinal imaging may detect systemic complications beyond that of DR, for example, imaging of arterioles and venules of the retina may serve as an indicator of circulatory disease [12], even with the use of AI techniques [13]. Deep learning–based algorithms implementing retinal images has been utilised with good performance in identifying patients with diabetic peripheral neuropathy (DPN) [14].

To date, no study has developed and validated AI based algorithm with the use of retinal images to diagnose CAN as well as to further differentiate between early (eCAN) and definite or severe involvement of CAN (dsCAN). Based on previous studies which proved efficient in identification of patients with CVD or DPN combining the retinal imaging with an AI-based deep learning algorithms we hypothesised that AI could be a potent tool to diagnose CAN in patients with DM.

## Methods

This is a single-centre, observational, study conducted in a cohort of patients with DM admitted to the Department of Internal Medicine and Diabetology, Clinic Hospital no 1 in Zabrze, Poland, and outpatients from the Diabetology Clinics in the Silesia Region, Poland, from October 6, 2021 until July 9, 2023 who fulfilled the inclusion and exclusion criteria. This is a part of the Silesia Diabetes-Heart Project (ClinicalTrials.gov Identifier: NCT05626413). Participant gave informed consent before any assessments took place. The study aligned with the Declaration of Helsinki standards, and was approved by the Ethics Committee of the Medical University of Silesia (KNW/0022/KB1/10/17).

### Inclusion and exclusion criteria

The following are the inclusion criteria for the study: (i) informed consent; (ii) diagnosis of T1DM for at least 5 years or T2DM for any duration prior to enrolment; (iii) age at least 18 years old. Exclusion criteria consisted of: proliferative retinopathy, any severe and acute illness, disabled and bedridden patients, solid organ transplant, previously diagnosed causes of neuropathy other than DM, pregnancy, alcohol use disorder, severe hypoglycaemia in the past 24 h.

### Medical history

Demographic data were reported by the patients and comorbid conditions were ascertained by documented medical history. Chronic kidney disease was defined as either estimated glomerular filtration rate (eGFR) < 60 ml/min per 1.73m^2^ or urine albumin to creatinine ratio (UACR) ≥ 30 mg/g for a duration of at least 3 months. DR grading was performed by experienced ophthalmologist based on fundus images collected in this study. Laboratory data were obtained during hospital stay or visit in outpatient clinic at the time of CAN and color fundus examination.

### CAN diagnosis

The CAN was diagnosed based on the Toronto consensus panel criteria with the use of cardiovascular autonomic reflex tests (CARTs), which are considered gold standard in CAN testing [2]. For CARTs assessment, DiCAN (Diabetic Cardiac Autonomic Neuropathy; Medicore, Seoul, South Korea) was utilized. Patients underwent CARTs in the morning (between 8:00 and 12:00 a.m.) in the same a consistently lit and quiet room. The heart rate responses to deep breathing test (expiration/inspiration ratio), lying-to-standing test (30:15 supine to standing ratio) and the Valsalva manoeuvre were evaluated. Furthermore, the assessment of orthostatic hypotension was performed by comparing blood pressure measurements before and after standing. If any of the CARTs tests results were missing, the patient was excluded from the analysis. The CAN was staged based on the number of abnormal CARTs results, using the following criteria: (i) early eCAN—one abnormal CART; (ii) definite CAN—at least two abnormal CARTs; (iii) severe CAN—at least two abnormal CARTs accompanied by orthostatic hypotension. Patients with advanced stages of CAN namely definite or severe CAN were grouped together for further analysis and labelled dsCAN. A detailed table describing the CARTs and their cutoffs for abnormal values has been displayed in Supplementary Table S1.

### Retinal images acquisition and processing

Retinal imaging was performed with the use of DRSplus device (Centervue, Padua, Italy) to capture five partially overlapping fields in each non-dilated eye, namely the central, nasal, temporal, superior, and inferior fields. This enabled an effective viewing angle of retina of up to 80 degrees. In the analysis, color fundus images were taken in individuals without CAN, with eCAN and with dsCAN. The study population was randomly split into training set (60%), validation set (10%) and test set (30%) on the patient-level to avoid data leakage.

### An exploratory analysis and model refinement

In an exploratory analysis, we initially incorporated retinal images from patients who had undergone laser therapy. It became evident, that the inclusion of these post-therapeutic images introduced a degree of noise into the diagnostic model. To enhance the model’s performance, these patients were subsequently excluded from the analysis (eight participants). We have performed final AI analysis utilising binary models in the two experiments. One of them aimed to differentiate patients with any stage of CAN from those without CAN and the second experiment aimed to differentiate patients with dsCAN from the group of patients with eCAN or no CAN.

### AI model—multiple instance learning

An AI based Multiple Instance Learning method (MIL) [15] was adopted for tackling the different number of colour fundus images per patient. Such MIL took all fundus images for each patient into account during AI model training, thus leading to a generalizable and explainable framework pipeline. MIL represents a type of weakly supervised learning where training examples are organized into groups known as “bags”, and a single label is assigned to the entire bag. In our context, we define a “bag” as a collection of multiple colour fundus images belonging to a single patient, and each individual image within the bag is referred to as an “instance,” following the conventional MIL framework. We align the label of the bag with the labels of its constituent instances, meaning that all instances within the same bag share the same label and are considered indicative of that label. However, it’s worth noting that since some colour fundus images may lack the distinctive features necessary for the backbone models to extract features and accurately predict the patient’s classification, there is a chance of introducing label noise, especially when considering negative bags. In such cases, it becomes imperative to incorporate the instances that truly contribute to the bag’s label, i.e., those instances whose actual hidden labels align with the bag’s true label. In this context, “discriminative” implies that an instance’s genuine, underlying label matches the actual label assigned to the bag. To address this, a robustness selection process is introduced into the MIL pipeline, where only the robust instances under adversarial noise’s perturbation are selected for feature learning in each model training epoch [15]. More details on the classic MIL algorithm, our proposed robustness-aware MIL process, the mathematical formulation, and the Shannon Entropy metric used for robustness quantification are provided in the Supplementary Material.

### Backbone networks

Different state-of-the-art AI backbone models are evaluated under the same experimental setting. For example, we adopted pre-trained cutting-edge classification backbone such as ResNet series (i.e. 18, 34, 50, 101), ResNext series (i.e. 50, 101), ResWide series (i.e. 50, 101), EfficientNet series (i.e. B0, B3, B5, B7), MobileNet series (i.e. V1, V2, V3), RegNet series (i.e. 16, 32) and Vision Transformer series (i.e. B16, B32, L16, L32, H14). A classification head is then added including a fully connected layer (1000 × 512), a ReLU layer, a dropout layer with 0.3 dropout rate, and a fully connected layer (512 × 2) in the end for outputting the final binary prediction.

### Implementation detail

The original fundus image size was 3600 × 2910 pixels; a bilinear interpolation resize operation was applied to resize the image into 224 × 224 pixels for efficient model training. An on-the-fly data augmentation method was adopted, specifically, we adopted random rotations and horizontal/vertical flips to the training data with a probability of 0.3. These rotations ranged from − 30 to 30 degrees. The neural network underwent end-to-end training for 400 epochs, starting with a learning rate of 1e-4 and following a cosine decay schedule. We utilized the Adam optimizer with a batch size of 48 and employed the standard Cross Entropy loss function to optimize the entire training process. All training procedures were carried out on a workstation equipped with four GEFORCE RTX 3090 24GiB GPUs. Subsequently, all testing experiments were performed on a local workstation featuring an Intel(R) Xeon(R) W-2104 CPU and a Geforce RTX 2080Ti GPU.

### Performance metrics and attribution maps

To assess the classification performance of the model, various test accuracy statistics were employed, including sensitivity, specificity, F1 score, precision, and the receiver operating characteristic (ROC) curve alongside the area under the ROC curve (AUROC). The precision in our findings was bolstered through the computation of 95% confidence intervals; specifically utilizing De Long’s method for AUROC and utilizing a 2000 sample bootstrap approach for determining the confidence intervals for sensitivity, specificity, F1 score, and precision.

In this study, we implemented the Grad-CAM attribution technique, which leverages the gradients directed into the last convolutional layer to construct a primary attribution map. This map highlights the crucial areas in the image predominantly influencing the classification outcome [16]. To augment this, we combined Grad-CAM with fine-grained details of the image, to get a Guided Grad-CAM. This refined visualization method enabled the creation of high-resolution, class-discriminative visualizations, shedding greater light on the elements vital for classification.

## Results

**Fig. 1:**
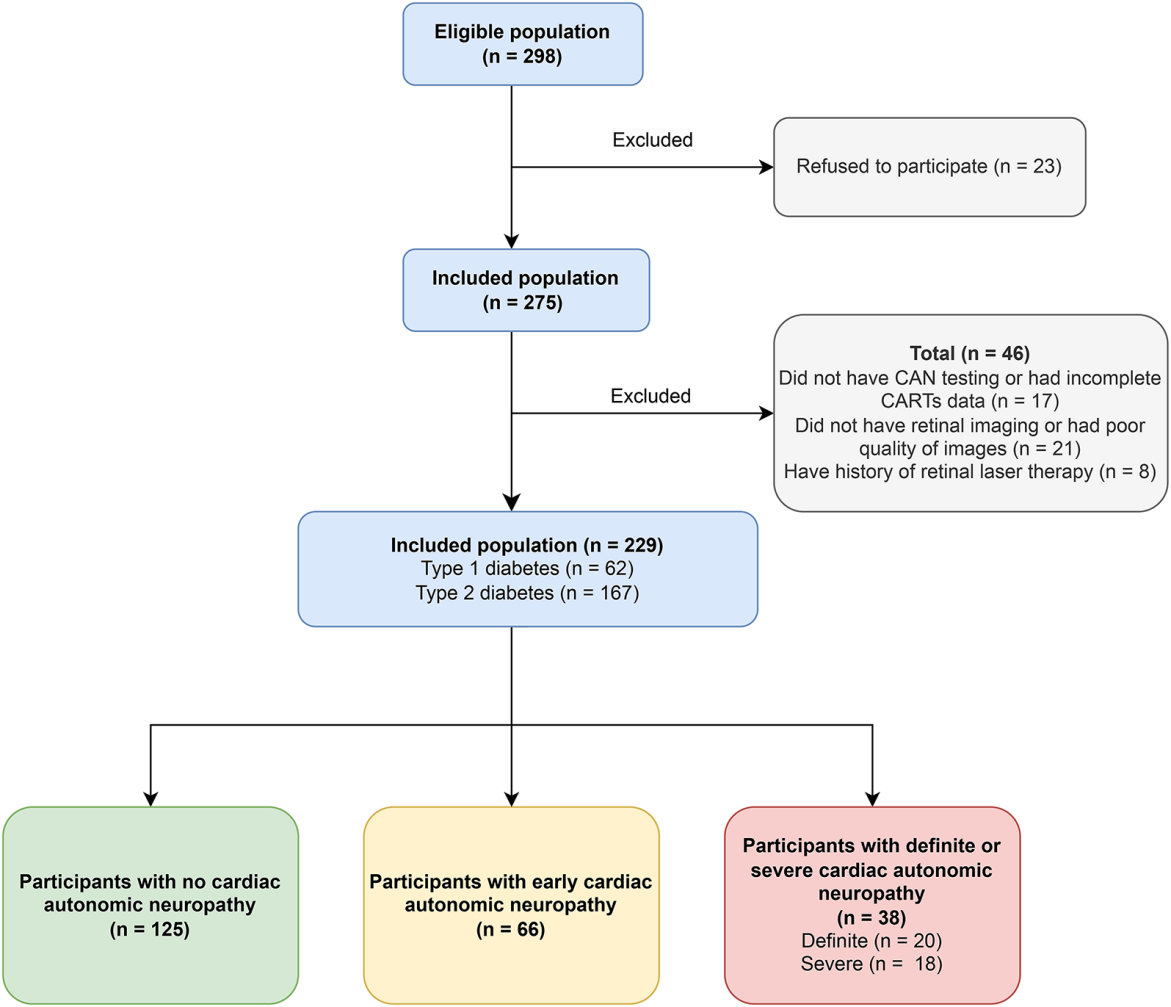
Flowchart of study participants. *CAN* Cardiac autonomic neuropathy, *CARTs* Cardiovascular autonomic reflex tests

From a primary eligible population of 298 patients, a total of 229 patients were included in the final analysis. The reasons for exclusion were the lack of consent for fundus and CAN examination (n = 23), no retinal imaging (n = 21), missing or incomplete CAN testing (n = 17). Patients with a history of retinal laser therapy (n = 8) were excluded after initial analysis. The study flowchart is presented in Fig. 1. Among analysed patients, 109 (45%) presented with any stage of CAN of whom 38 (17%) were diagnosed with dsCAN and 66 (29%) with eCAN. Demographic and clinical characteristics of studied participants are summarised in Table 1. Patients diagnosed with CAN were older, predominantly with type 2 DM and had a higher number of comorbidities. Patients with advanced stages of CAN had a longer duration of DM with higher rate of coronary artery disease and lower eGFR. The distribution of participants and images into training, validation, and test sets, along with a breakdown by CAN status, is delineated in Supplementary Tables S2 and S3, respectively.

**Table 1:** Baseline characteristic of study participants

Characteristic	No CAN N = 125^a^	eCAN N = 66^a^	dsCAN N = 38^**a**^	p-value^b^
Male	59 (47%)	30 (45%)	19 (50%)	> 0.9
Age, years	48 (28, 60)	61 (53, 69)	66 (60, 70)	< 0.001
BMI, kg/m^2^	27.6 (23.7, 31.7)	29.5 (25.8, 32.2)	30.7 (28.1, 35.8)	0.004
Type of diabetes				< 0.001
1	49 (39%)	13 (20%)	0 (0%)	
2	76 (61%)	53 (80%)	38 (100%)	
DM duration	10.0 (5.0, 14.0)	9.0 (4.0, 12.0)	15.0 (10.0, 19.8)	0.002
Diabetic retinopathy	35 (28%)	18 (27%)	10 (26%)	0.8
Mild	20 (16%)	9 (14%)	4 (11%)	
Moderate	12 (9.6%)	6 (9.1%)	5 (13%)	
Severe	3 (2.4%)	4 (6.1%)	1 (2.6%)	
Chronic kidney disease	32 (26%)	24 (36%)	15 (39%)	0.14
Hypertension	62 (50%)	49 (74%)	29 (76%)	< 0.001
Coronary artery disease	6 (4.8%)	7 (11%)	11 (29%)	< 0.001
History of stroke	7 (5.6%)	3 (4.5%)	1 (2.6%)	> 0.9
History of myocardial infarction	6 (4.8%)	6 (9.1%)	6 (16%)	0.077
Atrial fibrillation	8 (6.4%)	4 (6.1%)	5 (13%)	0.3
HBA1C, %	7.6 (6.7, 9.2)	8.0 (6.6, 10.4)	8.1 (7.2, 9.4)	0.2
UACR, mg/g	8.1 (5.0, 15.5)	8.2 (6.8, 33.2)	15.3 (6.5, 32.8)	0.13
eGFR, mL/min/1.73m^2^	94.8 (78.8, 106.4)	92.2 (74.4, 102.0)	75.4 (55.8, 87.7)	< 0.001
Total cholesterol, mmol/L	4.7 (4.1, 5.5)	4.7 (4.0, 5.9)	4.5 (3.8, 5.0)	0.3
LDL, mmol/L	2.5 (2.0, 3.1)	2.4 (1.8, 3.5)	2.2 (1.5, 2.7)	0.15
Triglycerides, mmol/L	1.2 (0.8, 1.9)	1.4 (1.0, 1.9)	1.8 (1.4, 2.2)	0.008
Antihypertensive drugs use	55 (44%)	46 (70%)	29 (76%)	< 0.001
ACEi	28 (22%)	32 (48%)	16 (42%)	< 0.001
ARB	13 (10%)	9 (14%)	8 (21%)	0.2
Beta blocker	36 (29%)	31 (47%)	18 (47%)	0.017
CARTs				
Deep breathing test	0 (0%)	42 (64%)	36 (95%)	< 0.001
Valsalva test	0 (0%)	11 (18%)	21 (57%)	< 0.001
30:15 test	0 (0%)	13 (21%)	14 (38%)	< 0.001
Orthostatic hypotension test	19 (15%)	15 (23%)	18 (49%)	< 0.001

*ACEi* angiotensin converting enzyme inhibitor, *ARB* angiotensin receptor blocker, *BMI* body mass index, *CAN* cardiac autonomic neuropathy, *CARTs* cardiovascular autonomic reflex tests, *eCAN* early CAN, *dsCAN*definite or severe CAN, DM diabetes mellitus, *eGFR* estimated glomerular filtration rate, *LDL* low density lipoprotein, *UACR* urine albumin creatinine ratio

^a^n (%); Median (IQR)

^b^Pearson’s Chi-squared test; Kruskal–Wallis rank sum test; Fisher’s exact test

### Model performance for the classification of CAN

**Fig. 2:**
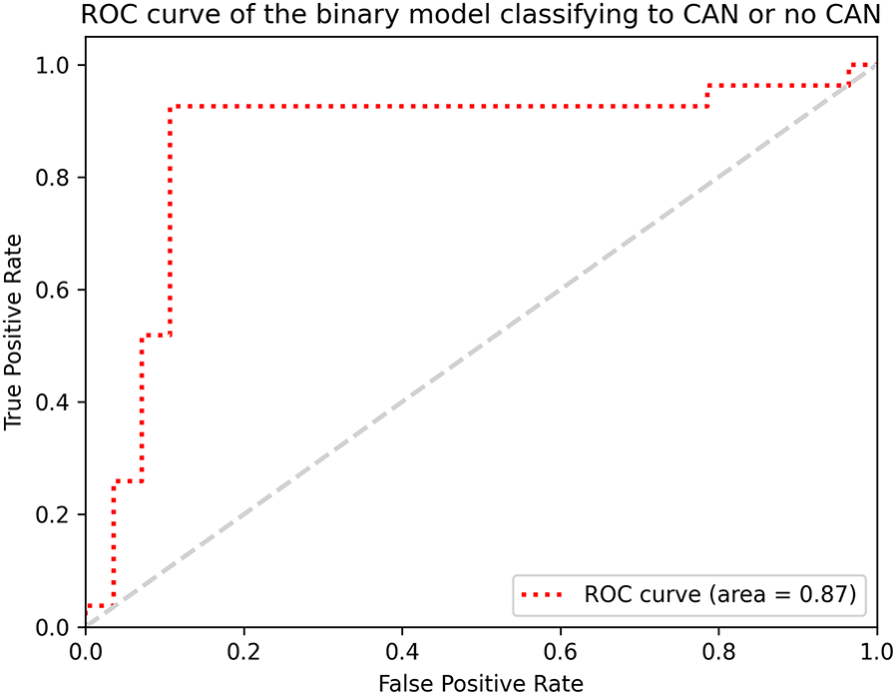
ROC curve of the binary model classifying to CAN or no CAN

For the binary classification to either no CAN or any stage of CAN (early, definite or severe), ResNet 18 achieved the best performance overall, correctly classifying 25 out of 27 (93%) patients with CAN and 25 out of 28 (89%) without CAN within the test set. Confusion matrix is shown in Table 2 and performance metrics of the model are reported in the Table 3. The model achieved an AUCROC of 0.87 (95% CI 0.74–0.97) (Fig. 2). Performance of the models utilizing alternative network backbones can be found in the Supplementary Material (Supplementary Table S4 and Figure S1).

**Table 2:** Confusion matrix for the ResNet 18 model differentiating patients with CAN and those without CAN

		Predicted class	
		No CAN	CAN
True class	No CAN	25	3
	CAN	2	25

*CAN* Cardiac autonomic neuropathy

**Table 3:** Performance metrics of the two experiments utilising binary models differentiating patients with any stage of CAN from those without CAN and those with eCAN or no CAN from those with dsCAN

Model	Sensitivity	Specificity	AUC	Precision	F1-score
ResNet 18 (CAN vs no CAN)	0.93 (0.82–1.00)^*^	0.89 (0.76–1.00)	0.87 (0.74–0.97)	0.89 (0.76–1.00)	0.91 (0.76–0.98)
ResNet 18 (dsCAN vs eCAN or no CAN)	0.78 (0.43–1.00)	0.93 (0.85–1.00)	0.94 (0.86–1.00)	0.70 (0.38–1.00)	0.74 (0.43–0.92)
ResWide 50 (dsCAN vs eCAN or no CAN)	0.89 (0.63–1.00)	0.91 (0.82–0.98)	0.91 (0.73–1.00)	0.67 (0.38–0.93)	0.76 (0.50–0.93)

*AUC* area under the curve, *CAN* Cardiac autonomic neuropathy, *CI* confidence interval, *dsCAN* definite or severe CAN, *eCAN* early CAN

^*^95% CI

### Model performance for the classification of definite or severe stages of CAN

**Fig. 3:**
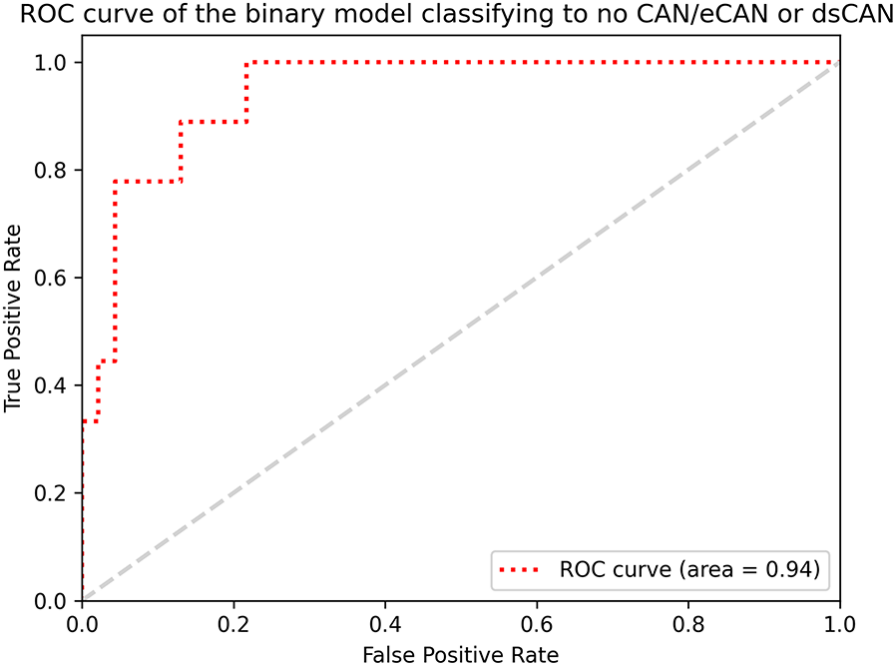
ROC curve of the binary model classifying to no CAN/eCAN or dsCAN

In the experiment of the binary classification which aimed at detecting dsCAN from those with eCAN or without CAN, the ResNet 18 model correctly identified 7 out of 9 (78%) patients with dsCAN and 43 out of 46 (93%) patients either without CAN or with eCAN in the test set. Table 4 outlines the confusion matrix, and the performance metrics can be reviewed in Table 3. The ResNet 18 model attained AUCROC of 0.94 (95% CI 0.86–1.00), as demonstrated in Fig. 3. Model based on the ResWide 50 achieved better sensitivity, correctly classifying 8 out of 9 patients (89%) with dsCAN, but with poorer AUCROC of 0.91 (95% CI 0.73–1.00) (Table 3). Performance of the alternative model backbones can be found in Supplementary Figure S2 and Table S5.

**Table 4:** Confusion matrix for the ResNet 18 model differentiating patients with dsCAN from those with eCAN or without CAN

		Predicted class	
		No CAN or eCAN	dsCAN
True class	eCAN or no CAN	43	3
	dsCAN	2	7

*CAN* cardiac autonomic neuropathy, *dsCAN* definite or severe CAN, *eCAN* Early CAN

### Attribution maps

**Fig. 4:**
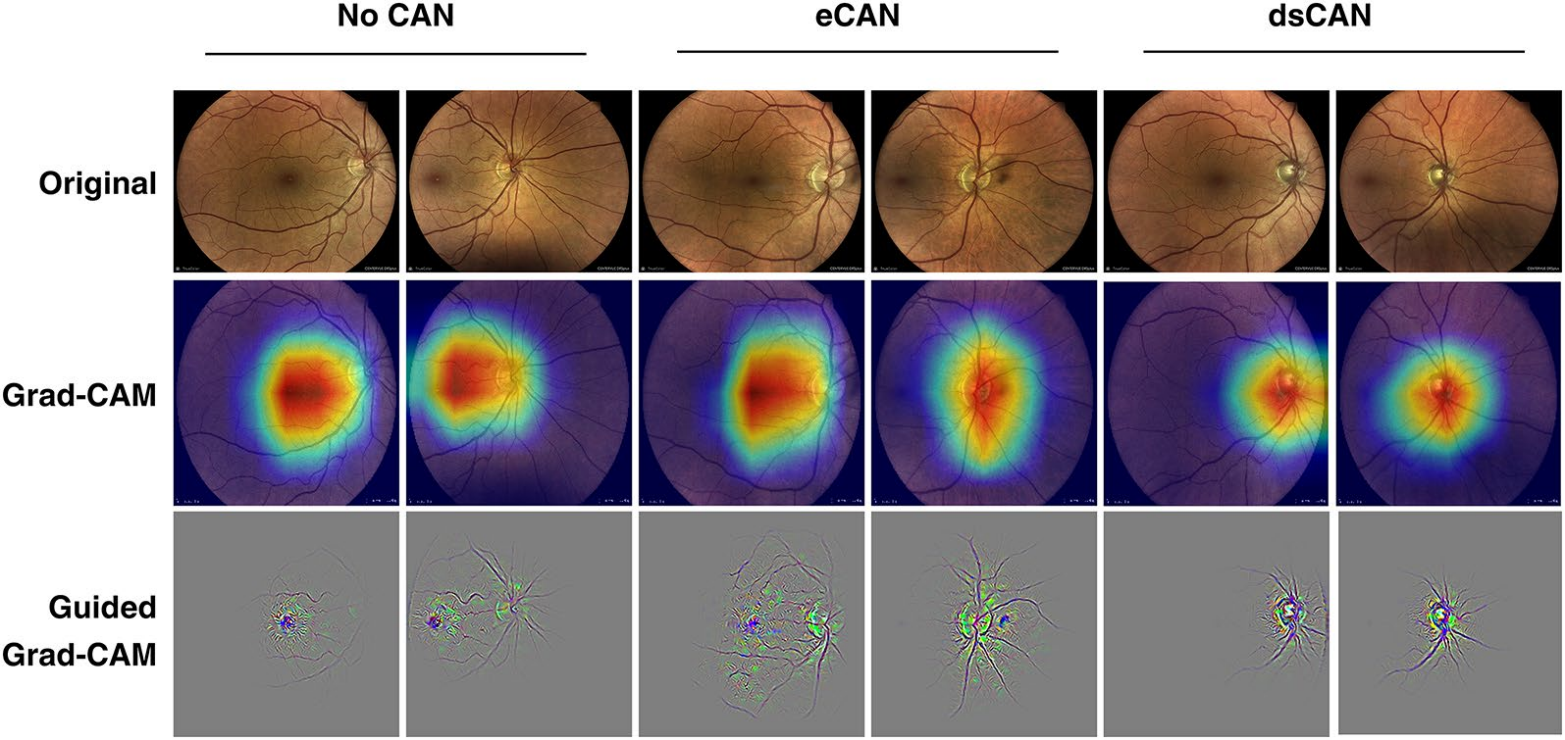
Attribution map results from ResNet 18. Example images from patients without CAN, with eCAN or dsCAN. First row, original images, second row, Grad-CAM images, third row, Guided Grad-CAM images

Figure 4 displays retinal images from the test set that were correctly identified by the ResNet 18 model, alongside the corresponding Grad-CAM and Guided Grad-CAM visualizations. The attribution maps for correctly classified patients without CAN highlighted the macula and optic disc. Conversely, in subjects with dsCAN, the optic disc was the primary focus of the attribution maps. Additionally, the Guided Grad-CAM visualizations were notable for their emphasis on the retinal vasculature, a feature that was more pronounced in the peripheral retinal fields, as presented in Supplementary Figure S3.

## Discussion

The main findings of our study are as follows: (i) The development of a novel AI-based deep learning algorithm(s) which detect CAN and classify its severity; (ii) Our binary AI algorithm achieving good sensitivity with excellent specificity for CAN detection; and (iii) a further AI-based deep learning algorithm (DLA) to detect severe CAN manifestations with high sensitivity and specificity. As far as we are aware, this is the first study that provides a solution to the problematic underdiagnosis of CAN in patients with DM with the use of AI-based DLA using the retinal fundus images.

Our AI model performs classification without the need for expert annotation, remedying operator bias and importantly utilising DR screening to diagnose a distinct microvascular complication which is not routinely screened. Importantly, previous research utilising clinical data demonstrates that AI using data driven machine learning approaches achieves outstanding performance for the prediction of CAN (AUC: 0.96 [95% CI 0.94–0.98]) and an accuracy of 87%, and sensitivity of 87% [17]. In the present study, using fundus imaging, our models had excellent diagnostic ability to identify patients with CAN (sensitivity 0.93, specificity 0.89, AUC 0.87) and those with definite or severe CAN presentations (sensitivity 0.78, specificity 0.93, AUC 0.94).

AI has been extensively researched in the detection of DR, specifically referrable given the current increase in the global prevalence of visual impairment and also the associated health economic costs of expert graders to identify early DR in retinal fundus images [18]. Indeed, AI-based algorithms were first utilized in US for DR screening and are established in the detection of referable DR [19]. Subsequently, preliminary AI-based algorithms have been developed with the aim to predict favorable outcomes with anti-VEGF injections [20]. Microangiopathy is a cardinal pathophysiological feature of DM complications including DR and DPN. As such vasculopathy of DR noted on fundus images may serve as a biomarker for risk stratification of other diabetes-related microvascular complications [21]. It has been demonstrated that retinal microvasculature analysis using AI predicts: (1) a number of CVD (cardiovascular disease) risk factors including DM and hypertension, (2) direct CVD events including CV mortality, and (3) CVD biomarkers such as coronary artery calcium score [22]. Recently, Mordi et al. [23] have demonstrated that retinal parameters alone and in combination with genome-wide polygenic risk score for coronary heart disease have independent and incremental prognostic value compared with traditional CV risk assessment in T2DM.

Lee et al. [24] successfully presented AI-based models which predicted the risk of DPN through the development of four deep learning architectures (InceptionNet, VGGNet, ResNet, and ConvMixer) using fundus photography images. The combined sensitivity values of disease severity stratified DPN reached 0.84, 0.90, 0.90, and 0.92 (mild-moderate-severe) and thus demonstrated that the AI-based models were able to determine the presence of DPN and its associated severity [24]. We have previously demonstrated the use of AI to diagnose DPN through the use of corneal nerve images. In a seminal study, Williams et al. [25] segmented corneal nerves from corneal confocal microscopy images and used U-Net models to demonstrate superior segmentation and classification performance compared to other non-AI based automated models. Subsequently, Preston et al. [9] developed a DLA using ResNet backbone architecture to detect peripheral neuropathy (in DM and prediabetes) and similar to the current study it achieved excellent classification accuracy using end-to-end classification (without the need for segmentation). We further developed this model into a clinically translatable binary classification system of no DPN vs DPN present [26].

Color fundus photography is an established, readily available diagnostic tool primarily deployed for DR screening. The American Diabetes Association (ADA) advocates for biennial screening for individuals with DM who exhibit no signs of DR and endorses the use of retinal photography in screening programs [27]. This established infrastructure for fundus imaging presents a favourable framework for extending its utility to the detection of additional diabetic complications, offering a cost-effective approach without necessitating supplementary testing procedures. Our AI-driven model processes multiple retinal images per subject, encompassing the peripheral retinal fields, which may capture nuanced vascular changes indicative of systemic complications. The rationale for using retinal imaging in conjunction with DLA for the purpose of neuropathy diagnosis is underpinned by findings from Neriyanuri et al. [28] Their study revealed that DPN manifests in the retina as structural and functional impairments, despite the absence of DR. This is characterized by increased foveal thickness and reduced retinal nerve fibre layer thickness, alongside declines in various visual functions. Choi et al. [29] further support this by demonstrating significant associations between inner retina thickness and cardiovascular autonomic dysfunction. Their study found that eyes with retinal nerve fibre layer defects had significantly thinner ganglion cell-inner plexiform layer thicknesses, correlating with early and definite stages of CAN. These retinal changes suggest the existence of subtle structural alterations that could be detected by sophisticated DLAs. It is well recognized that diabetic microvascular disease complications cluster, and as such, CAN is more likely to occur when diabetic retinopathy is present. Notably, our study demonstrated no differences in rates of DR across patients without CAN and differing severity of CAN. Thus, suggesting that retinal changes associated with neuropathies may have distinctive features independent of the classic DR markers.

While the subtle structural changes related to CAN are not readily discernible to human clinicians through traditional fundus examination, our study illustrates the potential of AI in unveiling these hidden patterns. There are no direct visual features for diagnosing CAN through retinal images in current clinical practice. However, the high accuracy of our AI models suggests that these computational techniques can identify minute retinal alterations beyond human visual capability. Previous research has also effectively utilized machine learning methodologies in conjunction with fundus photography to yield appreciable diagnostic accuracy for DPN. For instance, Benson et al. employed a support vector machine classifier, attaining a sensitivity of 78% and a specificity of 95% in identifying DPN [30].

We applied the Toronto criteria for the diagnosis of CAN, which are based on CARTs and recognized as a gold-standard in CAN diagnostics [2]. This ensures a robust diagnostic framework and strengthens the validity of our model’s capability to accurately identify CAN. The present investigation utilized a modestly sized cohort (N = 229), which, while leading to wide CIs, still attained reasonable level of accuracy in classification. Augmenting the model with a clinical and demographic data could potentially enhance its diagnostic precision and facilitate the creation of a three-tier classification system that would detect CAN and differentiate its severity within one model. Validation of this AI-driven DLA is necessary in a larger cohort and, subsequently, prospectively within a broader clinical setting. Upon successful validation, it will be crucial to develop cost-effectiveness frameworks to evaluate the potential economic implications of its healthcare application.

## Limitations

The study included a relatively small number of patients but remains a novel proof of concept for a wider clinical research. This was also a single centre study which requires further validation in a more heterogeneous populations of patients to test its performance in real-world clinical deployment. Future modification of the model by including clinical and demographic data may improve the diagnostic performance although currently achieved excellent classification accuracy. In contrast to conventional AI model training approaches for 2D images, the Multiple Instance Learning mechanism typically requires a longer duration for training. For instance, when considering a relatively limited training dataset, our AI model necessitates an average of 48 h for 400 epochs of training using various backbones. Nonetheless, we consider the training process for the model as a one-time event, whereas the speed of inference holds greater significance in assessing the algorithm’s performance and its applicability in real-world scenarios. Remarkably, our model achieves an inference time of approximately 0.19 s per patient on average, producing accurate diagnostic results.

In conclusion, AI-based algorithms utilising retinal images can differentiate with high accuracy patients with CAN. AI analysis of fundus images to detect CAN may be implemented in routine clinical practice to identify patients at the highest CV risk, however external validation of our findings and algorithm optimization in a prospective clinical study are required.

### Supplementary Information

Supplementary file 1 (DOCX 911 kb)

## Data Availability

After publication of the manuscript, data will be available at reasonable request from the corresponding authors after secondary analysis and development of subsequent algorithms.

## Abbreviations

ACEi: Angiotensin converting enzyme inhibitor
AI: Artificial intelligence
ARB: Angiotensin receptor blocker
AUC: Area under the curve
AUCROC: Area under the receiver operating characteristic curve
BMI: Body mass index
CAN: Cardiac autonomic neuropathy
CARTs: Cardiovascular autonomic reflex tests
CI: Confidence interval
CV: Cardiovascular
CVD: Cardiovascular disease
DiCAN: Diabetic cardiac autonomic neuropathy
DLA: Deep learning algorithm
DM: Diabetes mellitus
DPN: Diabetic peripheral neuropathy
DR: Diabetic retinopathy
dsCAN: Definite or severe CAN
eCAN: Early CAN
eGFR: Estimated glomerular filtration rate
LDL: Low density lipoprotein
MIL: Multiple Instance Learning
ROC: Receiver operating characteristic
T1DM: Type 1 diabetes mellitus
T2DM: Type 2 diabetes mellitus
UACR: Urine albumin creatinine ratio

## References

[1] IDF Diabetes Atlas 10th edition scientific committee. International Diabetes Federation. IDF Diabetes Atlas, 10th edn. Brussels, Belgium: 2021. https://www.diabetesatlas.org. IDF official website. 2021.

[2] Spallone V, Ziegler D, Freeman R, Bernardi L, Frontoni S, Pop-Busui R, et al. Cardiovascular autonomic neuropathy in diabetes: clinical impact, assessment, diagnosis, and management. Diabetes Metab Res Rev. 2011;27:639–53. 21695768 10.1002/dmrr.1239

[3] Martin CL, Albers JW, Pop-Busui R. Neuropathy and related findings in the diabetes control and complications trial/epidemiology of diabetes interventions and complications study. Diabetes Care. 2014;37:31–8. 24356595 10.2337/dc13-2114 PMC3868000

[4] Low PA, Benrud-Larson LM, Sletten DM, Opfer-Gehrking TL, Weigand SD, O’Brien PC, et al. Autonomic symptoms and diabetic neuropathy: a population-based study. Diabetes Care. 2004;27:2942–7. 15562211 10.2337/diacare.27.12.2942

[5] Maser RE, Mitchell BD, Vinik AI, Freeman R. The association between cardiovascular autonomic neuropathy and mortality in individuals with diabetes: a meta-analysis. Diabetes Care. 2003;26:1895–901. 12766130 10.2337/diacare.26.6.1895

[6] Chowdhury M, Nevitt S, Eleftheriadou A, Kanagala P, Esa H, Cuthbertson DJ, et al. Cardiac autonomic neuropathy and risk of cardiovascular disease and mortality in type 1 and type 2 diabetes: a meta-analysis. BMJ Open Diab Res Care. 2021;9:2480. 10.1136/bmjdrc-2021-002480 PMC8719152 34969689

[7] Williams S, Raheim SA, Khan MI, Rubab U, Kanagala P, Zhao SS, et al. Cardiac autonomic neuropathy in type 1 and 2 diabetes: epidemiology, pathophysiology, and management. Clin Ther. 2022;44:1394–416. 36272822 10.1016/j.clinthera.2022.09.002

[8] Kumar Y, Koul A, Singla R, Ijaz MF. Artificial intelligence in disease diagnosis: a systematic literature review, synthesizing framework and future research agenda. J Ambient Intell Humaniz Comput. 2023;14:8459–86. 35039756 10.1007/s12652-021-03612-z PMC8754556

[9] Preston FG, Meng Y, Burgess J, Ferdousi M, Azmi S, Petropoulos IN, et al. Artificial intelligence utilising corneal confocal microscopy for the diagnosis of peripheral neuropathy in diabetes mellitus and prediabetes. Diabetologia. 2022;65:457–66. 34806115 10.1007/s00125-021-05617-x PMC8803718

[10] Rudnicka AR, Welikala R, Barman S, Foster PJ, Luben R, Hayat S, et al. Artificial intelligence-enabled retinal vasculometry for prediction of circulatory mortality, myocardial infarction and stroke. Br J Ophthalmol. 2022;106:1722–9. 36195457 10.1136/bjo-2022-321842 PMC9685715

[11] Zhou Y, Chia MA, Wagner SK, Ayhan MS, Williamson DJ, Struyven RR, et al. A foundation model for generalizable disease detection from retinal images. Nature. 2023;622(7981):156–63. 37704728 10.1038/s41586-023-06555-x PMC10550819

[12] Monteiro-Henriques I, Rocha-Sousa A, Barbosa-Breda J. Optical coherence tomography angiography changes in cardiovascular systemic diseases and risk factors: a review. Acta Ophthalmol. 2022;100:e1-15. 33783129 10.1111/aos.14851

[13] Poplin R, Varadarajan AV, Blumer K, Liu Y, McConnell MV, Corrado GS, et al. Prediction of cardiovascular risk factors from retinal fundus photographs via deep learning. Nature biomedical engineering. 2018;2:158–64. 31015713 10.1038/s41551-018-0195-0

[14] Cervera DR, Smith L, Diaz-Santana L, Kumar M, Raman R, Sivaprasad S. Identifying peripheral neuropathy in colour fundus photographs based on deep learning. Diagnostics (Basel, Switzerland). 2021;11:1943. 34829290 10.3390/diagnostics11111943 PMC8623417

[15] Meng Y, Bridge J, Addison C, Wang M, Merritt C, Franks S, et al. Bilateral adaptive graph convolutional network on CT based Covid-19 diagnosis with uncertainty-aware consensus-assisted multiple instance learning. Med Image Anal. 2023;84:102722. 36574737 10.1016/j.media.2022.102722 PMC9753459

[16] Selvaraju RR, Cogswell M, Das A, Vedantam R, Parikh D, Batra D. Grad-CAM: visual explanations from deep networks via gradient-based localization. Proceed IEEE Int Conf Comput Vis. 2017;2017:618–26.

[17] Abdalrada AS, Abawajy J, Al-Quraishi T, Islam SMS. Prediction of cardiac autonomic neuropathy using a machine learning model in patients with diabetes. Ther Adv Endocrinol Metab. 2022;13:20420188221086692. 35341207 10.1177/20420188221086693 PMC8943459

[18] Leasher JL, Bourne RRA, Flaxman SR, Jonas JB, Keeffe J, Naidoo K, et al. Global estimates on the number of people blind or visually impaired by diabetic retinopathy: a meta-analysis from 1990 to 2010. Diabetes Care. 2016;39:1643–9. 27555623 10.2337/dc15-2171

[19] Lim JI, Regillo CD, Sadda SR, Ipp E, Bhaskaranand M, Ramachandra C, et al. Artificial intelligence detection of diabetic retinopathy: subgroup comparison of the eyeart system with ophthalmologists’ dilated examinations. Ophthalmol Sci. 2023;3:100228. 36345378 10.1016/j.xops.2022.100228 PMC9636573

[20] Takahashi H, Tampo H, Arai Y, Inoue Y, Kawashima H. Applying artificial intelligence to disease staging: deep learning for improved staging of diabetic retinopathy. PLoS ONE. 2017;12:e0179790. 28640840 10.1371/journal.pone.0179790 PMC5480986

[21] Pearce I, Simó R, Lövestam-Adrian M, Wong DT, Evans M. Association between diabetic eye disease and other complications of diabetes: Implications for care. A systematic review. Diabetes Obes Metabol. 2019;21:467–78. 10.1111/dom.13550 PMC6667892 30280465

[22] Wong DYL, Lam MC, Ran A, Cheung CY. Artificial intelligence in retinal imaging for cardiovascular disease prediction: current trends and future directions. Curr Opin Ophthalmol. 2022;33:440–6. 35916571 10.1097/ICU.0000000000000886

[23] Mordi IR, Trucco E, Syed MG, MacGillivray T, Nar A, Huang Y, et al. Prediction of major adverse cardiovascular events from retinal, clinical, and genomic data in individuals with type 2 diabetes: a population cohort study. Diabetes Care. 2022;45:710–6. 35043139 10.2337/dc21-1124

[24] Lee T-F, Chiu C-M, Tseng C-D, Huang H-Z, Lin C-H, Lin G-Z, et al. Using deep learning models to predict the risk of peripheral neuropathy on diabetic patients. Res Square. 2023;42:747.

[25] Williams BM, Borroni D, Liu R, Zhao Y, Zhang J, Lim J, et al. An artificial intelligence-based deep learning algorithm for the diagnosis of diabetic neuropathy using corneal confocal microscopy: a development and validation study. Diabetologia. 2020;63:419–30. 31720728 10.1007/s00125-019-05023-4 PMC6946763

[26] Meng Y, Preston FG, Ferdousi M, Azmi S, Petropoulos IN, Kaye S, et al. Artificial intelligence based analysis of corneal confocal microscopy images for diagnosing peripheral neuropathy: a binary classification model. J Clin Med. 2023;12:1284. 36835819 10.3390/jcm12041284 PMC9963824

[27] ElSayed NA, Aleppo G, Aroda VR, Bannuru RR, Brown FM, Bruemmer D, et al. Retinopathy, neuropathy, and foot care: standards of care in diabetes—2023. Diabetes Care. 2022;46:S203–15. 10.2337/dc23-S012 PMC9810462 36507636

[28] Neriyanuri S, Pardhan S, Gella L, Pal SS, Ganesan S, Sharma T, et al. Retinal sensitivity changes associated with diabetic neuropathy in the absence of diabetic retinopathy. Br J Ophthalmol. 2017;101:1174–8. 28108570 10.1136/bjophthalmol-2016-309641

[29] Choi JA, Kim HW, Kwon J-W, Shim Y, Jee DH, Yun J-S, et al. Early inner retinal thinning and cardiovascular autonomic dysfunction in type 2 diabetes. PLoS ONE. 2017;12:e0174377. 28334035 10.1371/journal.pone.0174377 PMC5363937

[30] Benson J, Estrada T, Burge M, Soliz P. Diabetic peripheral neuropathy risk assessment using digital fundus photographs and machine learning. Proceed Annu Int Conf IEEE Eng Med Biol Soc EMBS. 2020;2020:1988–91. 10.1109/EMBC44109.2020.9175982 33018393

