# Supplementary material for: Artificial intelligence-based classification of cardiac autonomic neuropathy from retinal fundus images in patients with diabetes: The Silesia Diabetes Heart Study
Source: Cardiovasc Diabetol. 2024 Aug 10;23:296. doi: 10.1186/s12933-024-02367-z (PMC11316981; doi:10.1186/s12933-024-02367-z)
Supplement: Supplementary file 1 — (DOCX 911 kb) [file 12933_2024_2367_MOESM1_ESM.docx]

**Supplementary Material**

**Artificial intelligence – based classification of cardiac autonomic neuropathy from retinal fundus images in patients with diabetes - The Silesia Diabetes Heart Study**

***Classic Multiple Instance Learning (MIL)***

MIL is a form of weakly supervised learning where training instances are grouped into sets called bags, and a single label is assigned to each bag, which is then applied to all instances within the bag. According to conventional MIL terminology, we refer to the collection of color fundus images associated with a participant as a bag, and each individual image within the bag as an instance. All instances in the same bag share the same label and are considered discriminative. However, there is a possibility that some instances may be negative, introducing label noise into the positive bags. To validate the model, it is essential to use robust training samples. In this context, 'robust' means that the true hidden label of an instance matches the actual label of the bag in every aspect.

Let $X = \left\{ X_{1,}X_{2,}\ldots X_{N} \right\}$ as the dataset containing $N$bags. Each bag $X_{i} = \left\{ X_{i,1}, X_{i,2}, \ldots, X_{i,N} \right\}$ consists of $N_{i}$ instances, where $X_{i,j} = \left\{ X_{i,j}, y_{i} \right\}$ is the $j-th$instance, $y_{i}$ denotes its associated label in the $i-th$bag. Please note that, $N_{i}$ may differ due to a different number of colour fundus images for different patients. The label $Y_{i}$ of bag $X_{i}$ is given by:

$$Y_{i} = \left\{ \begin{aligned} 0 iff \sum y_{i} = 0 \\ 1 otherwise. \end{aligned} \right.$$

(1)

Generally, a MIL-based prediction model contains an appropriate transformation

$f$ and a permutation-invariant transformation $g$ . Thus, the MIL’s prediction for the bag $X_{i}$

is defined as:

$$P\left( X_{i} \right) = g\left( f\left( x_{i}, 1 \right), f\left( x_{i}, 2 \right),\ldots, f\left( x_{i}, N \right) \right).$$

(2)

Regarding the selection of $f$and $g$, there are generally two types. Firstly, an instance-based approach. $f$is an instance classifier that assigns a score to each instance, and $g$is a pooling operator, such as max-pooling, that combines the instance scores to obtain a bag score. Specifically, a 2D CNN was trained to predict the class probability of each instance. During training, a few instances with higher responses were selected and back-propagation performed. An iteration process was utilised, with a new set of discriminative instances until coverage. Secondly, embedding-based approaches: $f$is an instance-level feature extractor that maps each instance to an embedding; $g$ is an aggregation operator that generates a bag embedding from the instance embedding and outputs a bag score based on the bag embedding. The embedding-based approaches provide a bag score from a bag embedding supervised by the bag label. The discriminative and non-discriminative instances’ embeddings contribute differently to the overall bag prediction. However, it is typically more challenging to identify the discriminative instances that activate the classifier, compared with instance-based approaches.

***Issues of classical instance-based MIL***

These issues can be broadly divided into two categories. Firstly, the distribution of instances in positive bags can be extremely imbalanced. This is particularly problematic when only a small proportion of instances are positive, leading models to misclassify these positive instances as negative, especially when using a simple aggregation operator such as max-pooling. Due to the assumptions of MIL and the imbalance of instances in a bag, max-pooling may shift the classification decision boundary closer to the uncertain (rare) instances [1].

Secondly, all instances in the same bag share the same label and are considered discriminatory. This assignment can inevitably introduce label noise into positive bags since some slices may be negative. Given that the learning mechanism is weakly supervised, the model is prone to overfitting to noisy and uncertain instances, which results in poor generalizability in real-world clinical practice. Moreover, instances with high uncertainty disproportionately affect the classification space, making it challenging to generalize learned boundaries to new test examples [2].

***Robustness-aware MIL***

To address the issues of imbalanced instance distribution and overfitting, we specifically designed a robustness-aware selection module within the standard instance-based MIL model training pipeline. This module incorporates a robustness-aware supervision process.

Firstly, to quantify the reliability of each instance's prediction, we adopted Shannon Entropy [3] as the metric to measure the randomness of the information [4]. Formally, given a $C$dimensional softmax predicted class score $P_{x_{i,j}}^{\left( C \right)}$ from an input instance $x_{i,j}$, the randomness $I_{i,j}$ is defined as:

$I_{i,j} = - \sum_{c=1}^{C} P_{x_{i,j}}^{(c)} \odot log P_{x_{i,j}}^{(c)}$,

(3)

Where $⨀$ is Hadamard Product; $C$is the number of classes. In practice, we perform $T$times stochastic forward passes on each instance classifier under random dropout and Gaussian noise perturbated input for each input instance. Note that $T$is empirically set as 6 in this work. Therefore, under such self-ensemble mechanism, we obtain a set of softmax probability vectors: $\left\{ P_{x_{i,j}}^{t} \right\}_{t=1}^{T}$, then the mean predicted class score $\tilde{P}_{x_{i,j}}^{\left( C \right)}$ is given as:

$$\tilde{P}_{x_{i,j}}^{\left( C \right)} = \frac{1}{T} \sum_{t=1}^{T} P_{x_{i,j}}^{t}$$

(4)

Thus based on equation (3) we can obtain the randomness $\tilde{I}_{x_{i,j}}$ for input instance $x_{i,j}$ as:

$$\tilde{I}_{x_{i,j}}= - \sum_{c=1}^{C} \tilde{P}_{x_{i,j}}^{\left( C \right)} \odot\log\tilde{P}_{x_{i,j}}^{\left( C \right)}$$

(5)

With the quantified uncertainty $\tilde{I}_{x_{i,j}}$ for instance $x_{i,j}$, we normalize $\tilde{I}_{x_{i,j}}$ into $\left[ 0, 1 \right]$ then perform element-wise broadcasting multiplication between $\tilde{I}_{x_{i,j}}$ and softmax predicted class score $P_{x_{i,j}}^{\left( C \right)}$. In this way, randomness-weighted probability prediction $P_{\tilde{I}_{i,j}}^{\left( C \right)}$ for each instance $x_{i,j}$ is calculated as:

$$P_{\tilde{I}_{i,j}}^{\left( C \right)}= \tilde{I}_{i,j} \bigotimes P_{x_{i,j}}^{\left( C \right)}$$

(6)

Where $\bigotimes$ denotes the element-wise broadcasting multiplication. In other words, the operator $g$ in our Robustness-aware MIL will consider the reliability of each $f\left( x_{i,N_{i}} \right)$ in Equation (2), and only the trustworthy colour fundus are considered for the model to learn the features in each training epoch. The learnt classifier considers the randomness level of the instance predictions to re-adjust boundaries (i.e. providing more room to uncertain samples). This improves the robustness ability of the proposed model for either imbalanced instances or weakly supervised learning mechanisms.

**Reference:**

1. Li B, Li Y, Eliceiri KW. Dual-stream multiple instance learning network for whole slide image classification with self-supervised contrastive learning. Paper/Poster presented at: Proceedings of the IEEE/CVF Conference on Computer Vision and Pattern Recognition; 2021;

2. Aljathlany Y, Alamari K, Aljasser A, Alhelali A, Bukhari M, Almohizea M, Khan A, Alammar A. Comparison Between Mathematical and Software Calculation Methods for the Measurement of the Cross-sectional Area in Upper Airway Imaging. *Cureus*. 2019;11:e6106. doi: 10.7759/cureus.6106

3. Shannon CE. A mathematical theory of communication. *The Bell system technical journal*. 1948;27:379-423.

4. Shannon CE. A mathematical theory of communication. *ACM SIGMOBILE mobile computing and communications review*. 2001;5:3-55.

**Cardiovascular autonomic reflex tests**

**Supplementary Table S1.** CARTs employed in the study and criteria for abnormal results

| Test | Method | Definition of abnormal result |
| --- | --- | --- |
| Deep breathing test | Six deep breaths for 1 minute in sitting position after normalization of hemodynamic parameters | Difference between maximal and minimal heart rate of <10 beats/min |
| Valsalva test | Subject blowing into the mouthpiece for 15 s at expiratory pressure of over 40 mmHg in sitting position | The ratio between the longest and shortest RR interval <1.1 at 20 seconds or 60 seconds |
| 30:15 test (lying-to-standing) | Change of the position from supine to upright | The ratio of the R-R intervals at the 30th and 15th beats <1 |
| Orthostatic hypotension test | Change of the position from supine to upright | Reduction of the systolic blood pressure >20 mmHg within 3 minutes of standing |

**Dataset Allocation, Participant and Image Distribution**

**Supplementary Table S2. Allocation to Training, Validation, and Test Sets**

| Data Subset (split %) | Number of Participants | Number of Images |
| --- | --- | --- |
| Training Set (60%) | 140 | 1,391 |
| Validation Set (10%) | 33 | 325 |
| Test Set (30%) | 55 | 549 |

**Supplementary Table S3. Distribution of Images and Participants by CAN Status**

| CAN Status | Number of Participants | Number of Images |
| --- | --- | --- |
| No CAN | 125 | 1,244 |
| eCAN | 66 | 659 |
| dsCAN | 38 | 372 |
| Total | 229 | 2,275 |

CAN, cardiac autonomic neuropathy; eCAN, early CAN; dsCAN, definite or severe CAN

**Experiments with Alternative Model Backbones**

To provide a thorough evaluation, we expanded our testing framework to include other neural network architectures in addition to the ResNet 18 model reported in the main manuscript. In the Supplementary Table S2 and Supplementary Table S3 we present the highest-performing models within the ResNext, ResWide, EfficientNet, MobileNet, RegNet, and Vision Transformer series to provide more comprehensive overview.

Of note, for the classification to CAN or no CAN, RegNet 16 yielded robust performance, closely mirroring the effectiveness of the ResNet 18 model. It registered a sensitivity of 0.85 [95% CI, 0.71 - 0.97], and a specificity of 0.89 [95% CI 0.76 - 1.00]. The AUC score for RegNet 16 was 0.88 [95% CI, 0.77 - 0.97].

While the primary manuscript elaborates on the superior performance of the ResNet 18 model, these additional results with alternative neural network architectures further underscore the versatility of CAN diagnosis based on fundus images coupled with deep learning.

**Supplementary Table S4. Performance metrics of alternative model backbones for the classification to CAN or no CAN**

| Model | Sensitivity | Specificity | AUC | Precision | F1-score |
| --- | --- | --- | --- | --- | --- |
| ResNext 50 | 0.89 [0.76 - 1.00]* | 0.79 [0.63 - 0.93] | 0.88 [0.77 - 0.96] | 0.80 [0.65 - 0.93] | 0.84 [0.72 - 0.93] |
| ResWide 50 | 0.82 [0.67 - 0.96] | 0.79 [0.63 - 0.93] | 0.81 [0.68 - 0.92] | 0.79 [0.63 - 0.93] | 0.80 [0.67 - 0.91] |
| EfficientNetB0 | 0.93 [0.81 - 1.00] | 0.57 [0.38 - 0.76] | 0.80 [0.66 - 0.91] | 0.68 [0.52 - 0.83] | 0.78 [0.66 - 0.88] |
| MobileNet V3 | 0.74 [0.57 - 0.90] | 0.82 [0.67 - 0.95] | 0.85 [0.73 - 0.94] | 0.80 [0.64 - 0.95] | 0.77 [0.63 - 0.88] |
| RegNet 16 | 0.85 [0.71 - 0.97] | 0.89 [0.76 - 1.00] | 0.88 [0.77 - 0.97] | 0.88 [0.75 - 1.00] | 0.87 [0.76 - 0.95] |
| Vision Transformer B16 | 0.78 [0.61 - 0.92] | 0.89 [0.76 - 1.00] | 0.82 [0.70 - 0.93] | 0.88 [0.73 - 1.00] | 0.82 [0.69 - 0.93] |

* 95% CI

We investigated the performance of additional neural network architectures for the specific task of distinguishing more severe CAN presentations. The results presented in Supplementary Table S3 show findings for the best performing architecture in a series.

EfficientNetB3 showed exceptional specificity, recording a value of 0.98 [95% CI, 0.93 - 1.0]. However, its sensitivity was markedly lower at 0.22 [95% CI, 0.00 - 0.57], pointing to limitations in detecting dsCAN specifically. MobileNet V2 demonstrated a balanced performance with a sensitivity of 0.67 [95% CI, 0.29 - 1.0] and a specificity of 0.85 [95% CI, 0.73 - 0.95]. It achieved an AUC score of 0.83 [95% CI, 0.61 - 0.98], marking it as a reliable alternative for this classification task. ResNext 50, while not outperforming in any specific metric, exhibited a decent overall performance. It achieved a sensitivity of 0.56 [95% CI, 0.20 - 0.88], specificity of 0.85 [95% CI, 0.74 - 0.94], and an AUC of 0.86 [95% CI, 0.72 - 0.95].

**Supplementary Table S5. Performance metrics of alternative model backbones for the classification to dsCAN or no CAN and eCAN**

| Model | Sensitivity | Specificity | AUC | Precision | F1-score |
| --- | --- | --- | --- | --- | --- |
| ResNext 50 | 0.56 [0.20 - 0.88]* | 0.85 [0.74 - 0.94] | 0.86 [0.72 - 0.95] | 0.42 [0.13 - 0.71] | 0.48 [0.17 - 0.72] |
| EfficientNetB3 | 0.22 [0.00 - 0.57] | 0.98 [0.93 - 1.0] | 0.71 [0.47 - 0.91] | 0.67 [0.00 - nan] | 0.33 [0.14 - nan] |
| MobileNet V2 | 0.67 [0.29 - 1.0] | 0.85 [0.73 - 0.95] | 0.83 [0.61 - 0.98] | 0.46 [0.18 - 0.75] | 0.55 [0.24 - 0.76] |
| RegNet 16 | 0.56 [0.20 - 0.89] | 0.87 [0.76 - 0.96] | 0.78 [0.59 - 0.94] | 0.45 [0.17 - 0.75] | 0.50 [0.19 - 0.75] |
| Vision Transformer L16 | 0.67 [0.33 - 1.0] | 0.85 [0.73 - 0.94] | 0.79 [0.61 - 0.94] | 0.46 [0.19 - 0.75] | 0.55 [0.25 - 0.78] |

* 95% CI


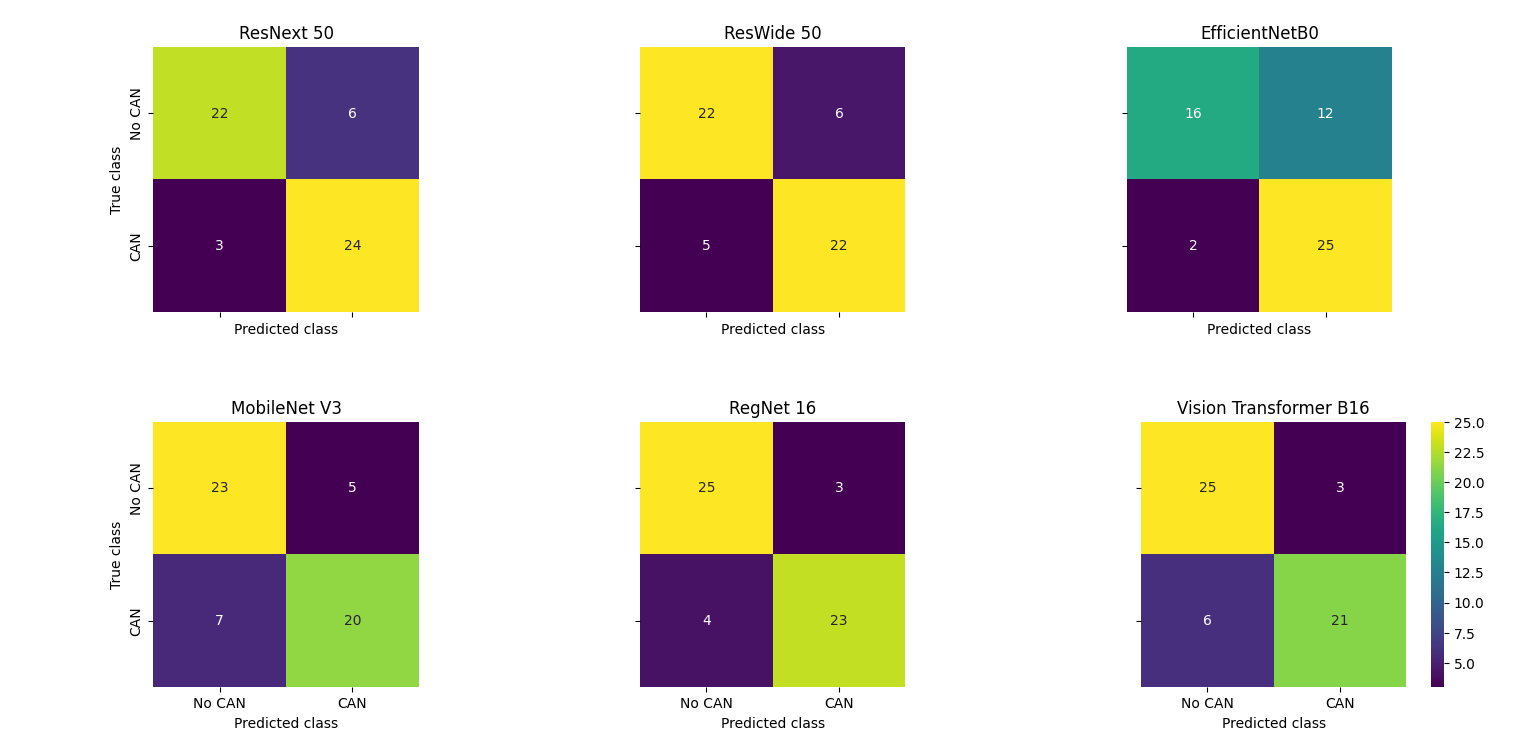


**Supplementary Figure S1. Confusion matrices of other backbones for classification of CAN vs no CAN**


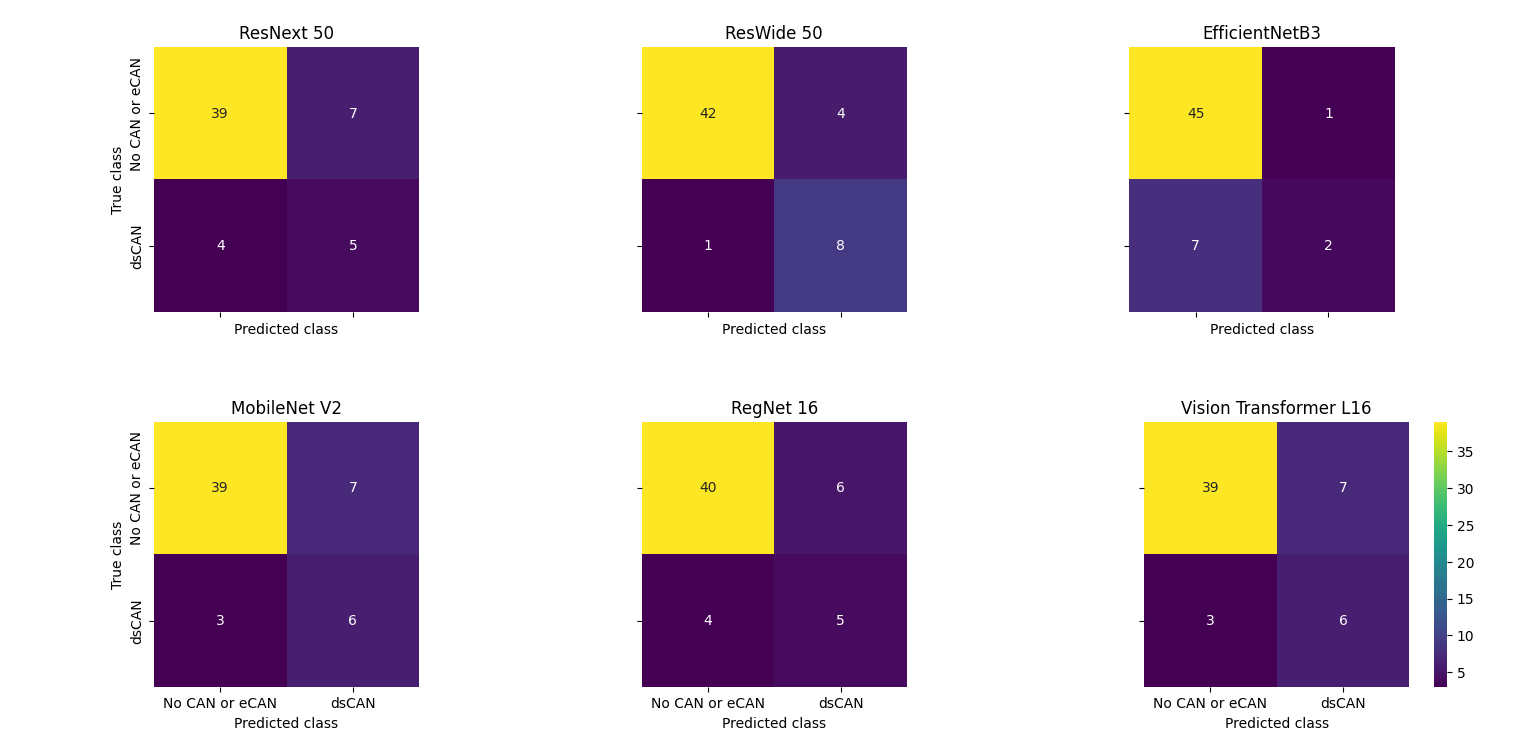
**Supplementary Figure S2. Confusion matrices of other backbones for classification of dsCAN**


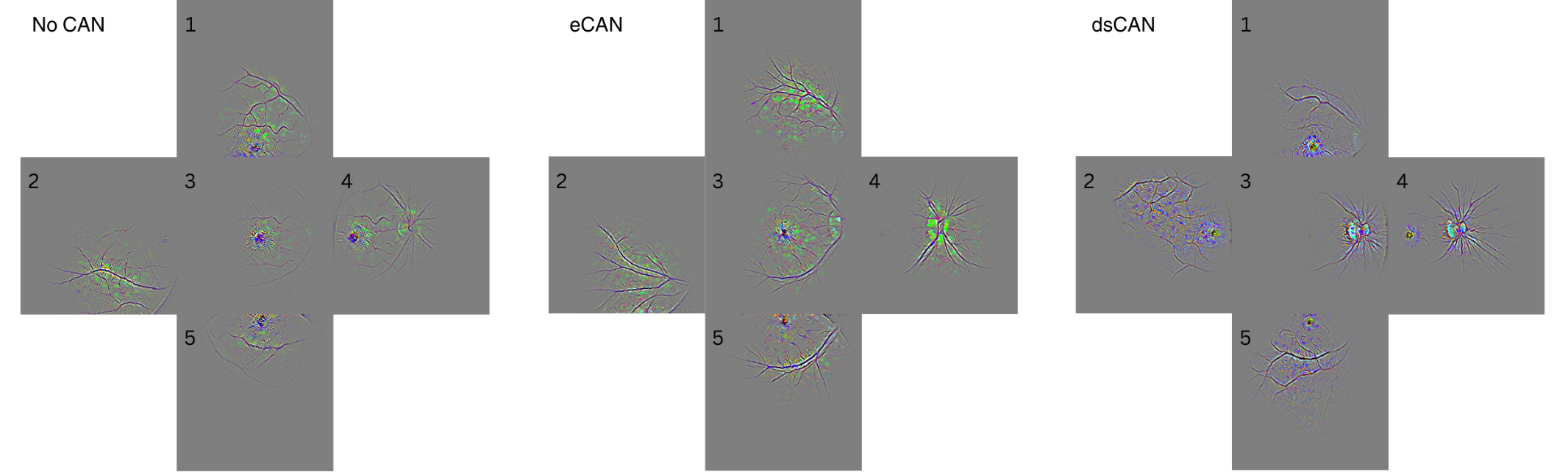


**Supplementary Figure S3. Guided Grad-CAM attribution maps results from ResNet 18. Example images from correctly predicted patients without CAN, with eCAN or dsCAN.** Images 1-5 are derived from different retinal fields of the same patient. 1, superior; 2, temporal; 3, central; 4, nasal; 5, inferior fields.
